# Protective activity of Panduratin A against Thioacetamide-induced oxidative damage: demonstration with *in vitro* experiments using WRL-68 liver cell line

**DOI:** 10.1186/1472-6882-13-279

**Published:** 2013-10-24

**Authors:** Suzy M Salama, Ahmed S AlRashdi, Mahmood A Abdulla, Pouya Hassandarvish, Mehmet Bilgen

**Affiliations:** 1 Department of Molecular Medicine and Biomedical Science, Faculty of Medicine, University of Malaya, 50603, Kuala Lumpur Malaysia; 2 Biophysics Department, Faculty of Medicine, Erciyes University, 38039, Kayseri, Turkey

**Keywords:** Cytotoxicity, Hepatocyte, Hepatoprotection, Boesenbergia rotunda, Panduratin A, WRL-68 liver cell line, Oxidative stress

## Abstract

**Background:**

Chalcone Panduratin A (PA) has been known for its antioxidant property, but its merits against oxidative damage in liver cells has yet to be investigated. Hence, the paper aimed at accomplishing this task with normal embryonic cell line WRL-68.

**Methods:**

PA was isolated from *Boesenbergia rotunda* rhizomes and its 2,2-diphenyl-1-picrylhydrazyl (DPPH) scavenging and ferric reducing power (FRAP) activities were measured in comparison with that of the standard reference drug Silymarin (SI). Oxidative damage was induced by treating the cells with 0.04 g/ml of toxic thioacetamide for 60 minutes followed by treatment with 1, 10 and 100 μg/ml concentrations of either PA or SI. The severities of oxidative stress in the control and experimental groups of cells were measured by Malondialdehyde (MDA) levels, superoxide dismutase (SOD), catalase (CAT) and glutathione peroxidase (GPx) activities.

**Results:**

PA exhibited an acceptable DPPH scavenging and FRAP activities close to that of Silymarin. Treating the injured cells with PA significantly reduced the MDA level and increased the cell viability, comparable to SI. The activities of SOD, CAT and GPx were significantly elevated in the PA-treated cells in a dose dependent manner and again similar to SI.

**Conclusion:**

Collectively, data suggested that PA has capacity to protect normal liver cells from oxidative damage, most likely via its antioxidant scavenging ability.

## Background

*Boesenbergia rotunda* (BR) is a perennial herb belonging to family Zingberaceae and traditionally used as folk medicine for treating diseases such as stomach discomfort, dysentery and leucorrhea in Southeast Asia. Past research have revealed that BR extract has an anti-cancer, antibacterial and neuroprotective properties [1-4]. In our previous *in vivo* experimental study, we have demonstrated that BR extract is hepatoprotective in liver cirrhosis [2]. Treatment of thioacetamide-induced hepatotoxicity with the extract was shown to maintain normal hepatic architecture and liver function by slowing down the progressive liver damage. Panduratin A (PA) is one of the chalcones present in the BR extract [5] and exhibits anti-inflammatory [5], anti-oxidant [3], antibacterial [6], anti-dengue [7], anti-mutagenic [8] activities. Cheah et al. 2011 reported that 30 μM (12.1 μg/mL) dose of PA is cytostatic but not cytotoxic to normal embryonic cell line WRL-68 [9]. Based on these multifunctions of PA, it is plausible that our previous findings on the hepatoprotectivity of the BR extract against liver cirrhosis may in part be due to the presence of this compound in the extract. Therefore, this study was initiated to investigate the hepatoprotective activity of PA when administered alone, specifically in thioacetamide-induced cytotoxicity, with *in vitro* experiments using WRL-68 cell line. In the following, we summarize our experimental procedures, present results and discuss our findings in the context of this objective.

## Methods

### Isolation of the compound Panduratin A from Boesenbergia rotunda extract

Ethanol based extract was obtained from *Boesenbergia rotunda* rhizomes (BR) by following the procedures described in our previous paper [2]. Briefly, fresh BR rhizomes were purchased from a company (Ethno Resources Sdn Bhd, Selangor, Malaysia) and identified by comparing against the specimen deposited at the Harbarium of Rimba Ilmu (Voucher number KU0098, Institute of Science Biology, University of Malaya, Kuala Lumpur). After washing the rhizomes with tap water first and then rinsing with distilled water, they were sliced and dried for 10 days. The dry samples were finely grounded, and 100 g of the resulting powder was mixed in 1000 mL solution of 95% ethanol for 7 days at room temperature. The mixture was distilled, and dried at 40°C in an incubator for 3 days giving a gummy yield of BR extract at 9.49% (w/w). Next, PA was isolated from this product. Specifically, 2.67 g of the extract sample was dissolved in acetone (Merck AR Grade, Malaysia) and further mixed with 5 g of silica gel (Silicycle, Ultrapure Silica Gel, Canada). The final mixture was dried using a rotary evaporator. The resulting powder was subjected to column chromatography (CC) containing 47 g of silica gel. The fractionation/isolation step was based on gradient elution method [10] with the Hexane-Ethyl acetate (Fisher Scientific AR Grade, UK) solvent system. A total of 11 fractions afforded. A Thin Layer Chromatography (Merck, Silica gel 60 F254, Japan) of the 11 fractions versus PA standard (98% purity) was carried out at the ratio of 95:5. Fraction BR-3 containing PA (R_f_ 0.29) was dried using a rotary evaporator and weighed using analytical balance to obtain 0.767 g pure crystals of PA (96.6%) as confirmed by High Performance Liquid Chromatography (HPLC) and calculated by the following formula:

$$\begin{array}{l} \%\;\text{Purity}=\text{Peak}\;\text{Area}\;\text{of}\;\text{Panduratin}\;A/\\ \;\left(\text{Total}\;\text{Peak}\;\text{Area}-\text{Peak}\;\text{Area}\;\text{of}\;\text{Solvent}\right). \end{array}$$

The mass of the isolated PA was determined by LCMS using METLIN DATABASE.

### DPPH scavenging activity of Panduratin A

The antioxidant power of the isolated compound PA was examined first by using 2,2-Diphenyl-1-picrylhydrazyl (DPPH) stable free radical scavenging assay as described by Brand et al. [11], but with a minor modification. The test involved dissolving 1 mg of the compound in 1 ml dimethyl sulfoxide (DMSO) (Fisher Medical, UK) and then further diluting into three different concentrations 1, 10 and 100 μg/mL. In addition, ascorbic acid and Quercetin were utilized as antioxidant standards and Silymarin was used as an antioxidant reference drug [12]. Amount (5 μL) of the compound and the standard were mixed with 195 μL of DPPH in triplicate using DMSO as blank. The decrease in absorbance value was assessed at 515 nm for 2 hr with 20 min gaps. Any antioxidant donates an electron to DPPH and the usual purple hue seen for free DPPH radical decays to yield a change in absorbency. The radical scavenging activity was quantified using the equation:

$$\begin{array}{l} \%\;\text{of}\;\text{fundamental}\;\text{scavenging}\;\text{activity}\\ \;=\left(\text{Abs}\;\mathrm{blank}\;-\;\text{Abs}\;\text{sample}\right)\;\times 100/\text{Abs}\;\text{blank}. \end{array}$$

### Ferric reducing power (FRAP) of Panduratin A

The anti-oxidant power of PA was also determined using a test sensitive to its scavenging ability towards reactive oxygen species or reagents containing iron. In this regard, the ferric reducing anti-oxidant power (FRAP) of the isolated compound was determined using an assay by following the method described in [13], with a slight modification. The FRAP reagent was prepared by mixing 300 mM acetate buffer (3.1 mg sodium acetate/ml, pH 3.6), 10 mM 2,4,6-tripyridyl-S-triazine (TPTZ) (Merck, Darmstadt-Germany) solution [14] and 20 mM FeCl_3_.H_2_O (5.4 mg/mL). Panduratin A compound, and the standards; Ascorbic acid and Quercetin were dissolved in DMSO and sampled in amounts of 10 μL of 1 μg/mL along with 10 μL of 1 μg/mL silymarin and added into 300 μL of the reagent TPTZ separately in triplicate using DMSO as blank. The absorbencies of the resulting mixtures were read using ELISA reader (UV 1601 spectrophotometer, Shimadzu, Kyoto, Japan) at 593 nm wavelength at 0 minute and after 4 minutes. The readings from the compound and silymarin were compared against the standards; Ascorbic acid and Quercetin [15].

### In vitro experiments

*In vitro* experiments involved acquiring a hepatic cell line, exposing cells to different concentrations of PA to show that PA is nontoxic, then subjecting the cells to toxicity through thioacetamide (TAA) and finally demonstrating that PA is effective in preventing the death of cells from TAA toxicity.

### Cell culture

Human embryonic normal liver cell line (WRL-68) was originally acquired from American Type Culture Collection ATCC and cultured in our institute. The cells were grown and maintained at 37°C by a 5% CO2 incubator (NuAire Inc., Plymouth, USA). When needed for experiments, normal cells were seeded in 24-well plate with density 3000 cells/well in 200 μL of RPMI-1640 containing 10% FBS and 1% (v/v) penicillin-streptomycin remedy, and incubated at 37°C and 5% CO2 for 24 hours before starting the experimentation [16].

### Determination of the IC_50_ dose of thioacetamide

To induce oxidative damage, WRL-68 cells were subjected to a toxic insult by TAA exposure. TAA has a short half-life and is metabolized in the microsomes of hepatocytes [17], but induces cytotoxicity in normal cells [6,7]. IC_50_ denotes inhibition concentration, i.e., concentration of TAA that causes the death of 50 % of the cells. Three different concentrations 0.03, 0.04, 0.05 g/ml of TAA were prepared by dissolving its crystals in sterile distilled water. These three concentrations were selected based on the previous publication, but stated as g/mL, and indicate amounts which cause oxidative damage to the cells [18]. The cells in three of the four wells were exposed to these concentration amounts by delivery in 10 μl, as one concentration per well, separately. The unexposed cells in the fourth well again served as control. As shown in results section, 0.04 g/ml was determined as the optimum IC_50_ dose. The percentage of cell viability was calculated by the formula:

$$\begin{array}{l} \%\;\text{Cell}\;\text{Viability}=(\text{Absorbance}\;\text{of}\;\text{the}\;\mathrm{TAA}-\text{treated}\;\text{cells}/\\ \;\text{Absorbance}\;\text{of}\;\text{sterile}\;\text{distilled}\;\text{water}\\ \;-\text{treated}\;\text{cells})\times 100. \end{array}$$

### Effects of Panduratin A in TAA-cytotoxicity

The WRL-68 cells in three of the four wells were treated with 10 μL of the optimum IC50 dose 0.04 g/mL of TAA. The cells in the fourth well served as normal control group. The cells in all four wells were incubated for one hour and then PA was added in concentrations of 1, 10 and 100 μg/mL, as in the experimentation aimed at determining the cytotoxicity of isolated PA above. The cells were incubated for 24, 48 or 72 hours and their viabilities were determined similarly.

Silymarin (SI) is a purified extract and used widely as a supportive therapy for liver disorders such as cirrhosis, hepatitis and fatty acid infiltration due to alcohol or toxic chemicals [14]. We also compared the effects achieved with PA on the TAA-cytotoxicity against the benchmark protection provided by SI [14,19]. The concentrations of SI were kept the same as those of PA. Concentrations of 1, 10 and 100 μg/mL of SI were respectively delivered to the cells in three separate wells, each in triplicate and their viabilities were evaluated at either 24, 48 or 72 hours [20].

### Assessment of MDA Level, CAT, SOD and GPx Activities

The following experiments were conducted to demonstrate the oxidative damage in the cells treated with the PA + TAA or SI + TAA compared to those treated with TAA only through the measurements in the levels of MDA and antioxidant enzymes (CAT, SOD and GPx) [14,19]. The cells were seeded and incubated first for one hour with 0.04 g/mL TAA and then treated with compounds PA or SI (1, 10, 100 μg/mL) as mentioned previously. After 72 hours of incubation at 37°C and 5% CO2, all cells were washed twice with 300 μl Phosphate Buffered Saline (PBS), pH 7.4. The cells were detached using a sterilized scrapper and lysed in 25 mmol/L Tris–HCl lysis buffer with no protease or phosphatase inhibitors used. The homogenate was thensonicated on ice (10 s pulse) and finally centrifuged at 13000 xg for 15 minutes. The resulting cell lysate was collected and kept at −20°C for assaying CAT, SOD, GPx and MDA following the instructions in the kit manuals of Cayman kits, USA (Cat. # 707002 for CAT, # 706002 for SOD, # 703102 for GPx and # 10009055 for MDA). Protein concentration in the cell lysate was evaluated using a standard method [21].

### Statistical analysis

All outcomes including the measured concentrations of the proteins CAT, SOD, GPx and MDA were evaluated statistically and the final results were expressed as Mean ± SEM (standard error in the mean) while n = 3. The data were analyzed by one-way analysis (ANOVA) followed by Tukey Post-Hoc test using SPSS (Version 18, SPSS Inc., Chi town, IL, USA). The level of significance was set at *p <* 0*.*05.

## Results

### HPLC and LCMS of PA

**Figure 1:**
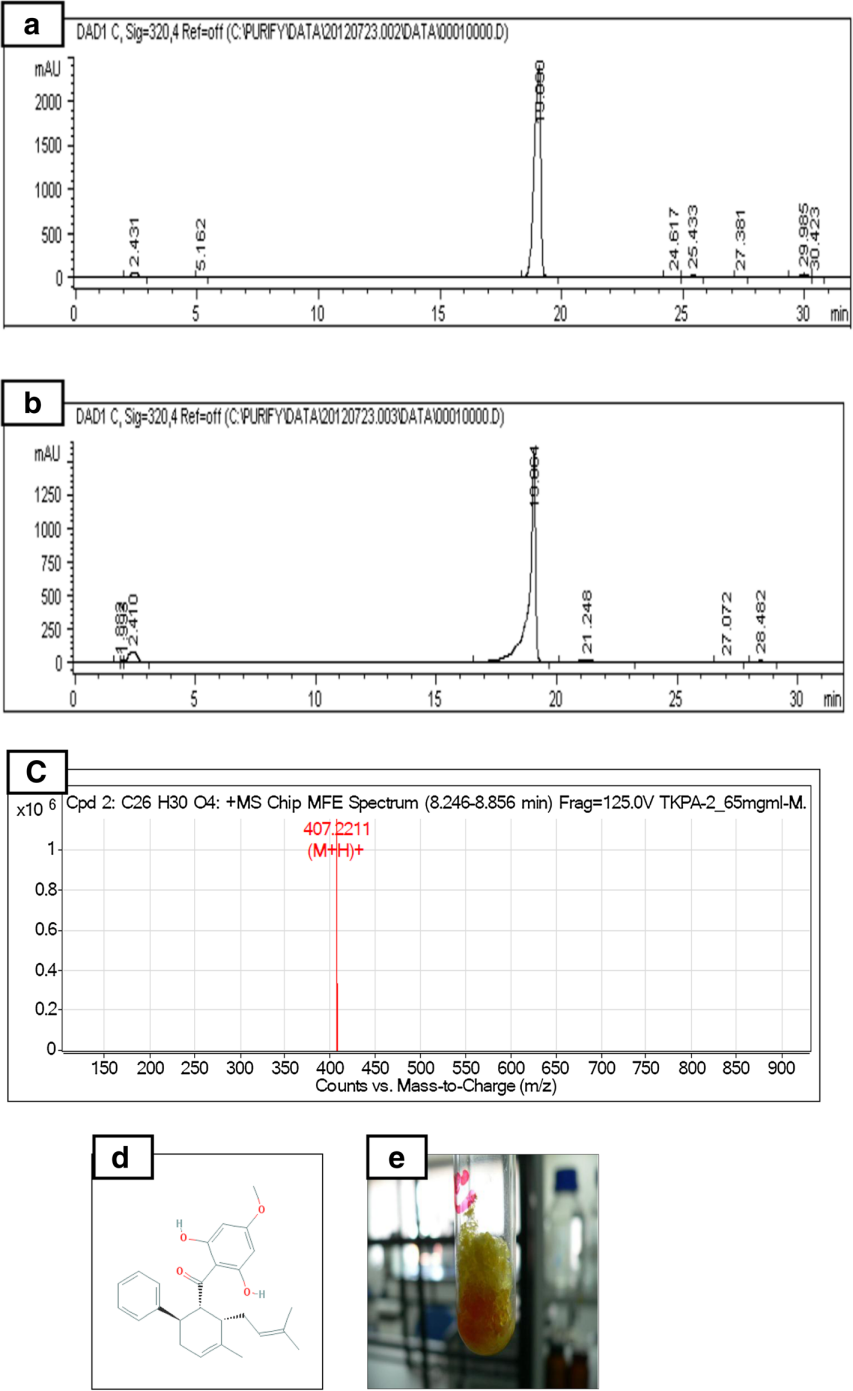
**Isolation of Panduratin A compound from the ethanol extract of *Boesenbergia rotunda *rhizomes. (a)** HPLC profile of BR-3 showing Panduratin A compound. **(b)** HPLC profile of Panduratin A standard. **(c)** Mass spectrum of Panduratin A. **(d)** Panduratin A (C_26_H_30_O_4_) structure. **(e)** Pale yellow crystals of isolated Panduratin A.

Dry pack column chromatography of *B. rotunda* crude extract resulted in 11 fractions. The results of TLC of all fractions versus PA compound showed that fraction BR-3 contained panduratin A recording retardation factor similar to that of PA standard (R_f_ = 0.29). These results were also confirmed by High performance liquid chromatography (Figure 1a and b). LCMS chromatogram of the isolated PA is illustrated in Figure 1c showing that the compound was detected at m/z 407.2211.

### DPPH scavenging activity

**Figure 2:**
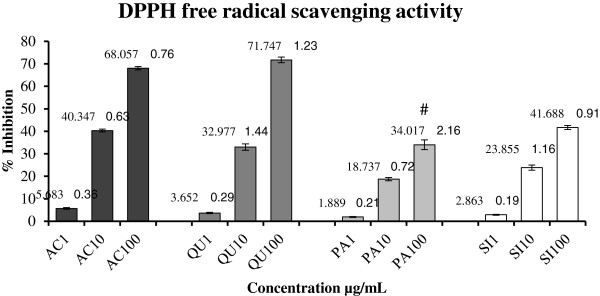
**Percentage inhibition of DPPH scavenging activity of Panduratin A compound (PA) compared with the standards Ascorbic acid (AC) and Quercetin (QU) and the reference drug Silymarin (SI).** Values are expressed as Mean ± SEM, while n = 3 *P<0.001 compared to AC100 and QU100. ^#^*P*<0.01 compared to SI100.

Panduratin A compound exhibited comparable free radical scavenging activity for DPPH compared to that of Quercetin (QU) and Ascorbic acid (AC) as shown in (Figure 2) (standard curve equation: y = 1.02333× – 0.1305, R^2^ = 0.9925). At the highest tested concentrations 100 μg/mL, the DPPH inhibition % of PA was significantly (*P* < 0.001) lower than that of the standards AC and QU and the reference drug Silymarin. On the other hand, at the lowest tested concentration 1 μg/mL, there was no significance between PA and the standards AC and QU or between PA and the standard drug SI. The medium concentration 10 μg/mL revealed low significance (*P* < 0.001) between PA and the standards AC and QU, while the significance detected between PA and SI was *P* = 0.52. These results indicated that PA had less potent radical scavenging effect and antioxidant activity than AC and QU, but similar to that of the reference drug SI at the low and medium concentrations 1 and 10 μg/mL, but not at the high concentration 100 μg/mL.

### FRAP value of PA

**Figure 3:**
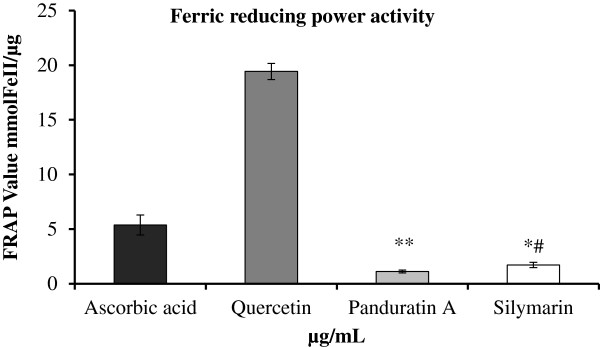
**Ferric reducing power (FRAP) of The isolated Panduratin A compound compared with the standards; Ascorbic acid, Quercetin as well as the standard drug Silymarin.** Values are expressed as Mean ± SEM, While n = 3 *P<0.01 compared to Quercetin. **P<0.01 compared to Ascorbic acid and Quercetin. ^#^*P*<0.05 compared to Ascorbic acid. No significance difference between Panduratin A and Silymarin.

As illustrated in Figure 3, the ferric reducing antioxidant power (FRAP) of Panduratin A was significantly lower (*P* < 0.01) (1.11 ± 0.13 mmol FeII/μg) than that of Ascorbic acid and Quercetin (5.37 ± 0.91 and 19.43 ± 0.74 mmol FeII/μg respectively), while the standard curve equation recorded y = 0.0006× + 0.624 and R2 = 0.9875. On the other hand, there was no significant difference between the measured FRAP value of Panduratin A and the standard drug Silymarin (1.72 ± 0.24 mmol FeII/μg). Further, the results revealed significant decrease in the FRAP value of Silymarin compared to Ascorbic acid (*P* < 0.01) and Quercetin (*P* < 0.05). Based on these readings, it is reasonable to extrapolate that Panduratin A exhibits an acceptable antioxidant activity similar to that of the standard drug Silymarin.

### In vitro protective activity of PA against TAA cytotoxicity

#### IC_50_ of TAA

**Figure 4:**
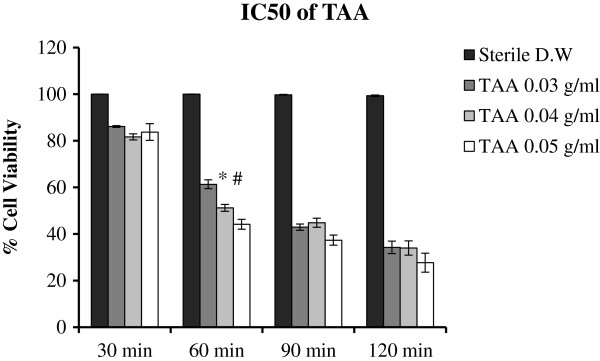
**IC**_**50 **_**of TAA. Data were expressed as Mean ± SEM, while n = 3.** *P<0.05 compared with TAA-treated cells (0.03 g/ml) and ^#^*P*<0.001 compared with TAA-treated cell (0.04 g/ml). D.W: distilled water; TAA: thioacetamide.

The percentage viability of WRL-68 cells after being incubated with different concentrations of TAA (0.03, 0.04 and 0.05 g/mL) for 30, 60, 90 and 120 min, are illustrated in Figure 4. Results showed that the IC_50_ dose of TAA is 0.04 g/mL at 60 min of incubation.

#### Protective effect of PA compound against TAA-toxicity

**Figure 5:**
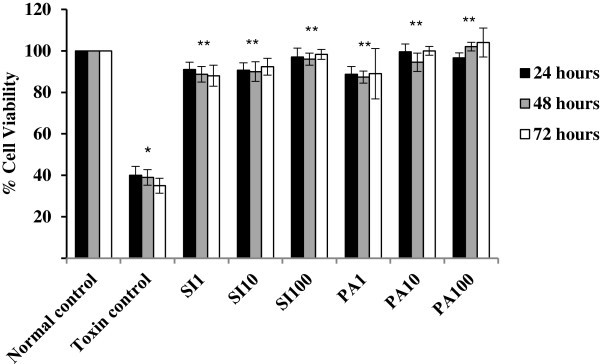
**The protective effect of Panduratin A (PA) against TAA toxicity in the WRL-68 cells.** Data were expressed as Mean ± SEM, while n = 3. Note that the data indicates % Cell variability exceeding 100 %, which means that PA not only protects the cells from the toxic effect of TAA, but also increases the cell proliferation to exceed the number of cells in the normal control.

The results of treating WRL-68 cells after being incubated for 60 minutes with TAA with three different concentrations of PA and SI compounds are shown in Figure 5. The data showed that, only about 35 % of the untreated cells remained alive at 24, 48 or 72 hours after being exposed to TAA. But, treatment of the cells with either PA or SI increased the cell survival significantly to about 90 % irrespective of the dose amount and the measurement time. No significant differences in the percentages of cell viabilities were recorded between the PA or SI treated cells indicating that PA had nearly the same protective effect as SI against TAA-cytotoxicity.

#### MDA and the antioxidant enzyme CAT, SOD and GPx assays

**Figure 6:**
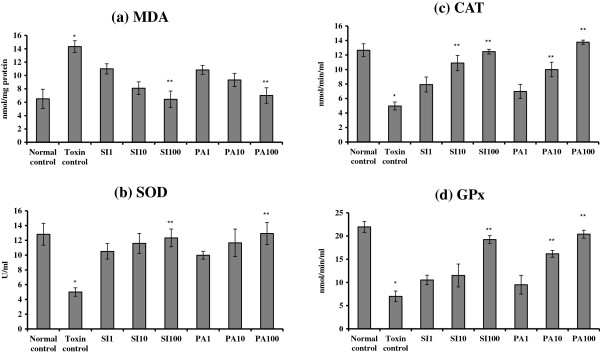
**The effect of Panduratin A (PA) treatment on oxidative stress and cellular antioxidant enzymes. (a)** MDA and the antioxidant activity **(b)** SOD,** (c)** CAT and **(d)** GPx of TAA-induced oxidative damage in WRL-68 cell line. WRL-68 cells were treated with TAA (0.04 g/mL) for 60 minutes followed by the addition of 1, 10 and 100 μg/mL of the mentioned PA extract and the SI compound. Data were expressed as Mean ± SEM, while n = 3. *P< 0.01 compared to normal control and **P<0.05 compared to toxin control.

As shown in Figure 6, treating WRL-68 cells with 0.04 g/mL TAA for 60 minutes markedly elevated the MDA protein level to 14.33 ± 0.88 nmoL/mg protein and reduced the antioxidant enzymes CAT, SOD and GP× activities to 4.97 ± 0.55 nmoL/min/mL, 5.00 ± 0.58 U/mL and 7.00 ± 1.15 nmol/min/mL, respectively compared to normal control cells (MDA: 6.50 ± 1.44 nmoL/mg protein, CAT: 12.67 ± 0.88 nmoL/min/mL, SOD: 12.83 ± 1.48 U/mL and GPx: 21.97 ± 1.18 nmoL/min/mL). PA treatment was effective towards the restoration of these biomarkers to their normal levels, as the dose increased. The highest dose 100 μg/mL of PA attenuated the protein MDA level back to 7.00 ± 1.15 nmoL/mg protein and increased the activities of antioxidant enzymes CAT, SOD and GPx to 13.77 ± 0.30 nmoL/min/mL, 12.93 ± 1.51 U/mL and 20.40 ± nmol/min/ml, respectively. There was no significant difference between the results obtained from treating the cells with either PA or SI at identical doses, indicating that PA was as effective as SI in protecting the cells against oxidative stress.

## Discussion

Liver cirrhosis is becoming more prevalent in today’s modern life as the health medications with side effects are increasingly being used. Consequently, current research targets new counteractive pharmaceutical solutions to tackle with this serious clinical problem [22]. Natural compounds or products extracted from folk medicinal plants are specifically investigated as potential therapeutic sources [23]. Efforts have been focused on evaluating their protective functions, especially with antioxidative impact, and also mechanisms of action in living model systems.

Phenolic substances present in the vegetation are regarded as nutritional antioxidants [24]. Chalcones are groups of phenolic compounds from the flavonoid family and exhibit a range of bioactivities, including anticancer [25] and antioxidative [24]. The anti-oxidant action of chalcones is relevant to various mechanisms such as quenching singlet oxygen, hydrogen atom or electron transfer and act as substrate for superoxide and hydroxide radicals. Anto et al. [26] tested 33 chalcones and chalcone related compounds and reported that most chalcones have high superoxide radical scavenging activity and Yayli et al. [27] confirmed these results. In the present study, the procedure of isolating PA from the ethanolic extract of *B. rotunda* rhizomes could isolate highly purified crystals of the compound and the quantity produced indicates that PA constitutes about 29% of the plant extract showing acceptable antioxidant %. DPPH as a stable free radical with structure containing an odd electron, is commonly used in chemical analysis procedures to measure free radical scavenging activity of compounds [28]. Panduratin A is one of the natural chalcones found in *Boesenbergia rotunda* rhizome extract and it’s previously demonstrated favorable biological activities made it attractive for investigating its merits as a potential therapy for the treatment liver cirrhosis in this research. Our results revealed that PA acquired an acceptable % DPPH inhibition activity (Figure 2) and ferric reducing power (Figure 3) when compared to the standard reference drugs. This was in line with the known property, i.e. high free radical scavenging activity, of chalcones [29].

Examining the cytotoxicity of Panduratin A on WRL-68 cell line revealed that PA was not cytotoxic even at the highest concentration of 100 μg/mL. This was in agreement with the findings from PA tests on different cell lines [3] where it was shown that 50 μM (20.3 μg/mL) of PA was not toxic.

TAA induces the production of reactive oxygen species (ROS) through its hydrolysis producing H_2_S as one of the reaction products [30]. Additionally, inside liver cells, H_2_S cytotoxicity elevates the level of ROS which are normally produced during cellular respiration depleting the level of antioxidant enzymes [31]. but, isolated compounds from plant extracts can effectively protect the hepatocytes from TAA-cytotoxicity [2,18]. Studies with cultured cells have proven the participation of oxidative stress in the etiology of TAA-induced oxidative damage [14,19]. TAA triggers fat peroxidation [32]; increases the vulnerability of hepatocytes to fat peroxidation [33]; and decreases the amount of endogenous anti-oxidant enzymes [34]. We determined that IC50 dose of TAA was 0.04 g/mL at 60 min of incubation with normal hepatocytes (Figure 4). PA treatment increased the viability of the TAA-exposed cells, implicating the protective activity of PA against TAA-cytotoxicity (Figure 5) which can be attributed to PA scavenging activity to ROS.

The hepatoprotective action was also confirmed by the levels of MDA and antioxidant enzymes (CAT, SOD and GPx) (Figure 6). As shown in Figure 6A, the high level of MDA in the cell lysate collected from the toxin control indicated that TAA triggered oxidative damage to cell membranes of liver cells. Fat peroxidation was started by reactive oxygen species (ROS) damaging the unsaturated fatty acids and spread by a chain reaction cycle including fats, lipid hydroperoxides and peroxy radicals [32] altering the cell membrane permeability and resulting in interruption in the structure and function of the membrane. The results of the present study suggest that panduratin A is able to reverse the hepatocyte lipid peroxidation brought on by TAA.

It is well recognized that cellular antioxdant enzymes, the most essential biomolecules defending against oxidative stress [35], can get involved in the decrease of sensitive intermediates. Anti-oxidants which can restrict the production of free radicals are essential with regards to defending the liver cells from toxin-induced damage by counterbalancing the cellular anti-oxidant systems. Whereas, some chalcones were found to inhibit glutathione and glutathione peroxidase (GPx) in hydrogen peroxide (H_2_O_2_) induced toxicity in liver cancer cells HepG_2_[36], but other chalcones were proved to increase the endogenous cellular enzymes SOD, CAT and GPx in Hydrogen peroxide-oxidative stress in neuroblastoma cells [37]. On the other hand, studies showed that chalcones have efficacy in inhibiting lipid peroxidation [30]. Our research revealed that TAA triggered ROS overproduction and decreased the cellular anti-oxidant enzymes SOD, CAT and GPx in the WRL-68 cells. The data further showed that SOD, CAT and GPx depletion due to TAA was recovered when the damaged cells were treated with panduratin A and the recovery was dose-dependent (Figure 6B, C and D). These positive results may be due to free-radical scavenging power of the chalcone PA as assisted by the percentage inhibition of DPPH scavenging activity of PA similarly to the reference drug Silymarin (Figure 2).

### Conclusion

Protecting hepatocytes *in vitro* from TAA-induced damage seems to be feasible when treated with Panduratin A. But, if the same would hold under *in vivo* conditions, i.e. if the liver’s structure and functions against toxins can be protected with PA, and if PA would play pharmacologic role in liver cirrhosis need further explorations.

## Competing interests

The authors declare that they have no competing interest.

## Authors’ contributions

SMS: Designing the research project, collection, analysis, and interpretation of data; writing of the manuscript and the decision to submit the manuscript for publication. AS: Interpretation of data and writing of the manuscript. MAA: Designing the research project and the decision to submit the manuscript for publication. PH: Collection and interpretation of the data. MB: Analysis and interpretation of data, writing and revision of the manuscript. All authors read and approved the final manuscript.

## Pre-publication history

The pre-publication history for this paper can be accessed here:

http://www.biomedcentral.com/1472-6882/13/279/prepub
